# Designing anti-epileptic drugs using neuronal dynamics

**DOI:** 10.1186/1471-2202-14-S1-P292

**Published:** 2013-07-08

**Authors:** Tyler Stigen, Theoden Netoff

**Affiliations:** 1 Biomedical Engineering, University of Minnesota, Minneapolis, MN 55455, USA

## 

While the molecular mechanism of many anti-epileptic drugs (AEDs) is known, rational drug design for anti-epileptic drugs has been hindered by our lack of understanding on how these mechanisms influence system level behaviors. Using phase reduction techniques, we can relate molecular level changes to network level synchrony to understand how a drug may influence system level behavior. After studying the effect of AEDs on neuronal dynamics using both electrophysiology and computational models, we observed a reduction in firing rate and minor changes in the phase response curve (PRC) to synaptic inputs. This finding was common among drugs with completely different mechanisms of action: phenytoin (voltage gated Na^+ ^blocker), ethosuximide (T-type Ca^++ ^blocker), and retigabine (voltage gated K^+ ^opener). When controlling for firing rate, we found no significant changes to the PRC of the neuron. The PRC relates the synaptic input to the spike time output of a neuron, it is logical that preservation of those dynamics is critical to the functional information processing in the system. This led us to hypothesize that the best AEDs may reduce the firing rate of the neuron while preserving the PRC. The slower firing rate reduces neurotransmitter release and synaptic summation, thus reducing activity in a population while maintaining the essential information transfer characteristics of the neuron. This hypothesis was tested in a computational neuron model [1], which has 6 currents and 36 parameters. A subset of parameters was examined, excluding those which would remain relatively static in a biological neuron. Each of the 24 qualifying parameters was altered until a 5 ms increase in the interspike interval was achieved compared to the control interspike interval (100 ms). We then measured the phase response curve and compared the sum squared error (SSE) relative to the control. Ranking the SSE of the PRC (Table 1) for each parameter showed that target of the known convulsant drug, 4-aminopyridine, had the greatest error in the phase response curve (156.3), while the known AED phenytoin showed lower error (115.9). Additionally, the highest ranking parameters (those which best preserve the PRC) were all K^+ ^currents that do not currently have a corresponding AED. Drugs targeting those parameters which best preserve the PRC while reducing firing rate may prove to be effective AEDs according to the hypothesis presented here. This technique is easily extensible to the addition of any channel and error rankings of the 24 parameters remained consistent across different firing rates. This method may provide a principled approach for determining which drug targets will be effective for the treatment of epilepsy and illustrate how neuronal dynamics can give us the information needed to understand system level effects related to molecular level drug mechanics, thus allowing us to engineer better medicine.

**Table 1:** Predicted Effectiveness of AED Target

**Parameter (Current)**	**PRC SSE**	**Parameter (Current)**	**PRC SSE**	**Parameter (Current)**	**PRC SSE**	**Parameter (Current)**	**PRC SSE**
theta nt (KDR)	10.8	sigma a (KA)	39.2	theta m (Na)	86.9	sigma n (KDR)	106.5
theta b (KA)	21.4	sigma nt (KDR)	41.2	sigma ht (Na)	90.2	theta p (NaP)^1^	108.4
theta a (KA)	22.4	sigma h (Na)	51.3	gL (Leak)	94.7	theta h (Na)^1,3-7^	115.9
gKA (KA)	26.1	theta ht (Na)	54.4	sigma p (NaP)	100.0	theta z (Kslow)	134.4
sigma b (KA)	28.4	gKSlow (Kslow)	74.8	sigma m (Na)	101.7	sigma z (Kslow)	138.9
gKDR (KDR)	38.1	gNa (Na)	82.0	gNaP (NaP)	102.7	theta n (KDR)^2^	156.3

Each parameter studied was ranked according the sum squared error of its measured PRC compared to the measured control PRC. Known convulsant drug, 4-aminopyridine (2), ranks last with the greatest error of the PRC, while known AED phenytoin (1) ranks higher. The top ranking currents are all associated with K^+ ^currents that currently have no associated AEDs, but in recent years K^+ ^channels have been seen as potential targets after the success of the drug retigabine. In this model, the K^+ ^M-current is part of the conglomerate Kslow current making commentary difficult. Other AEDs not examined in this study: valproate (3), topiramate (4), carbamazepine (5), oxcarbazepine (6), lamotrigine (7) also stabilize Na^+ ^inactivation.
